# Longitudinal observation of left ventricular inflow reorientation with preserved vorticity after myocardial infarction in a porcine model

**DOI:** 10.3389/fcvm.2026.1742432

**Published:** 2026-02-06

**Authors:** Sungho Park, Yura Ahn, Minseong Kwon, Hyun Jung Koo, Dong Hyun Yang, Hyungkyu Huh

**Affiliations:** 1 Medical Device Development Center, Daegu-Gyeongbuk Medical Innovation Foundation, Daegu, Republic of Korea; 2 Department of Radiology, Section of Pediatric Radiology, Children’s Hospital Colorado, University of Colorado Anschutz Medical Campus, Aurora, CO, United States; 3 Department of Radiology and Research Institute of Radiology, Cardiac Imaging Center, Asan Medical Center, University of Ulsan College of Medicine, Seoul, Republic of Korea

**Keywords:** cardiac magnetic resonance imaging, four-dimensional flow magnetic resonance imaging, left ventricular remodeling, myocardial infarction, rotational flow

## Abstract

**Background:**

The evolution of left ventricular (LV) intracardiac flow characteristics following myocardial infarction (MI), and their relationship to LV remodeling, remains incompletely understood, particularly from longitudinal *in vivo* observations with dense temporal sampling.

**Methods:**

Two porcine models with experimentally induced myocardial infarction at the left anterior descending artery were longitudinally followed from baseline to 11 weeks, with 10 cardiac MRI sessions per animal. Cardiac MRI assessed myocardial tissue characteristics (LGE, native T1/T2 mapping, and extracellular volume), global left ventricular function, and strain. Four-dimensional flow MRI was used to characterize intracardiac flow features, including E/A ratio, vorticity, and diastolic helical vortex structures. Longitudinal changes were examined descriptively within each animal. Overall temporal trends were summarized using the Theil–Sen estimator, with uncertainty assessed by residual bootstrap-derived 95% confidence intervals.

**Results:**

Both animals demonstrated overall remodeling patterns consistent with post-infarction progression, including temporal reductions in LGE with secondary changes during the chronic phase, thinning of the infarcted myocardial wall, and increases in indexed end-diastolic and end-systolic volumes. Native T1 relaxation time increased over time in one animal and showed a similar directional pattern in the other. Despite these structural and tissue-level changes, regional intracardiac vorticity remained relatively preserved throughout the study period. In contrast, the angle of the helical filling vortex between the anatomical LV and the vortex core centerlines was observed to increase in both animals.

**Conclusion:**

In this exploratory longitudinal study, both animals demonstrated similar temporal patterns of LV remodeling after MI, despite differences in the absolute magnitude of the measured parameters. In addition, our findings suggest that the angle of the helical filling vortex may have potential as a remodeling marker, which warrants validation in larger cohorts.

## Background

Myocardial infarction (MI) is closely associated with left ventricular (LV) remodeling, which develops in response to myocardial injury and increased wall stress (1, 2). This remodeling process can lead to pathological changes such as hypertrophy and ventricular dilation, thereby elevating the risk of arrhythmias and heart failure (HF) (3, 4). Echocardiographic assessment of LV function is widely recognized as a key component of routine evaluation in patients presenting with dyspnea or suspected HF (5). In particular, LV ejection fraction (EF) and global longitudinal strain (GLS) have been established as central biomarkers for clinical risk stratification (6–8). In a large consecutive cohort, 285 patients with MI demonstrated substantial impairments in LV EF and LV GLS during a 1-year follow-up period (9). Moreover, in a cohort of 1,409 patients with ST-segment elevation MI, a decrease in LV GLS over 1 year was associated with increased long-term all-cause mortality (10).

While echocardiography remains the gold standard for LV function assessment, cardiac magnetic resonance imaging (MRI) has emerged as reliable and reproducible structural characterization, particularly in evaluating LV remodeling after MI (11). For example, in patients with LV EF < 50% on echocardiography, subsequent MRI-based evaluation of LV EF has been shown to improve clinical decision-making and reduce major adverse cardiac events (12). In addition, cardiac MRI enables tissue characterization, including T1/T2 mapping and extracellular volume (ECV) quantification, which facilitates differentiation of the underlying etiology and stage of LV remodeling (13, 14). As such, timely and accurate cardiac MRI evaluation is therefore critical for detecting early myocardial injury during acute MI and preventing irreversible necrosis and maladaptive remodeling progression (15).

Recent advances in cardiac MRI enable time-resolved, phase-contrast, three-dimensional imaging of intracardiac blood flow, known as 4D flow MRI. This technique provides unique insights into LV function by allowing detailed flow analysis; for example, significant alterations in LV kinetic energy have been reported in both acute and chronic MI patients with preserved EF, compared to controls (16). In addition, late-diastolic kinetic energy has been independently associated with adverse LV remodeling (17). Metrics such as kinetic energy and local rotational flow vorticity are effective in identifying regions of stagnant flow in chronic MI, and abnormal inflow dynamics can lead to the formation of a tilted inflow vortex ring (18). These flow-based assessments provide complementary information to conventional echocardiographic and standard cardiac MRI measures, supporting the potential role of 4D flow MRI in post-MI prognostic evaluation.

However, most prior studies have been cross-sectional, reporting only low-to-moderate correlations between hemodynamic flow features and structural remodeling measures. More recently, a longitudinal study with three time points after acute MI (early post-MI, 3 months, and 12 months) demonstrated stronger associations between 4D flow parameters and myocardial strain, identifying direct flow as an independent predictor of adverse LV remodeling (19). Despite these advances, detailed longitudinal characterization of LV remodeling and intracardiac flow during MI progression remains limited, particularly due to sparse temporal sampling.

We hypothesized that a densely sampled longitudinal study would reveal stronger associations between structural remodeling and intracardiac flow features, and that progressive LV remodeling influences diastolic flow patterns. Accordingly, we performed cardiac MRI and 4D flow MRI to simultaneously assess myocardial tissue characteristics, LV function, and detailed intracardiac flow parameters, including the E/A ratio, vorticity (local rotational flow), and vortex-based flow organization.

## Methods

### MI-induced *in vivo* swine preparation

Two healthy porcine models (pig 1 and 2) were prepared after a period of acclimatization. A porcine model was anesthetized through an intramuscular injection of Zoletil (Virbac, France) at a dose of 5 mg/kg and Rompun (Bayer, Germany) at a dose of 2 mg/kg in a breeding room. The model was then taken to an imaging room, where it was administered inhalational anesthesia using Isoflurane, maintained at a concentration within the range of 1.2%–2.0% during the imaging. Physiological indicators such as oxygen saturation, heart rate, and electrocardiogram (ECG) were recorded and monitored. The femoral artery area was shaved, then disinfected three times with alcohol and povidone. To prevent inflammation and contamination during or after surgery, Tardomyocel compound (500 mL/bottle) was administered intramuscularly at a dose of 3 mL per 50 kg of body weight.

For an insertion of a coronary artery balloon catheter, the femoral artery was accessed using ultrasound guidance and a syringe on the sterilized groin area. Then, a guidewire was carefully inserted into the artery, and vascular imaging was conducted with biplane angiography equipment utilizing Iohexol (Omnipaque 240, GE Healthcare) at a total dose of 1–2 mL/kg to accurately position the guidewire in the ascending aorta. An arterial sheath (7 Fr) was then introduced, allowing the insertion of a catheter to perform an intervention on the mid left anterior descending (LAD) artery. The LAD was selected due to its higher likelihood of being linked to adverse clinical outcomes, as it supplies a greater portion of the myocardial territory compared to other coronary arteries (20, 21). The procedure involved inflating a balloon within the artery to a pressure of 8–12 atmospheres for approximately 30 s to experimentally simulate a MI scenario. This was followed by reperfusion, achieved by deflating the balloon catheter after an occlusion period of 1 h.

MRI examinations were conducted at following time points: baseline prior to MI (pre), immediately after (post), and then at 3 days (72 h), 1 week (1W), 2 weeks (2W), 3 weeks (3W), 4 weeks (4W), 5 weeks (5W), 7 weeks (7W), and 11 weeks (11W) after MI introduction. For each examination, the anesthesia protocol detailed previously was applied to ensure the porcine model's comfort and safety during the imaging process. After completion of the final MRI examination, the porcine models were euthanized in accordance with institutional guidelines. Animals were first deeply anesthetized with intramuscular Zoletil (5 mg/kg) and Rompun (2 mg/kg), followed by inhalational isoflurane at >5% to ensure loss of reflexes and deep anesthesia. Then, potassium chloride (KCl) was administered intravenously to induce cardiac arrest. The carcasses were subsequently stored in the laboratory's designated cold storage facility before being collected by a certified disposal company. All experimental procedures were approved by the Institutional Animal Care and Use Committee of The Laboratory Animal Center of the Daegu-Gyeongbuk Medical Innovation Foundation (IACUC; DGMIF-19120901).

### Cardiac MRI imaging protocol

**Figure 1:**
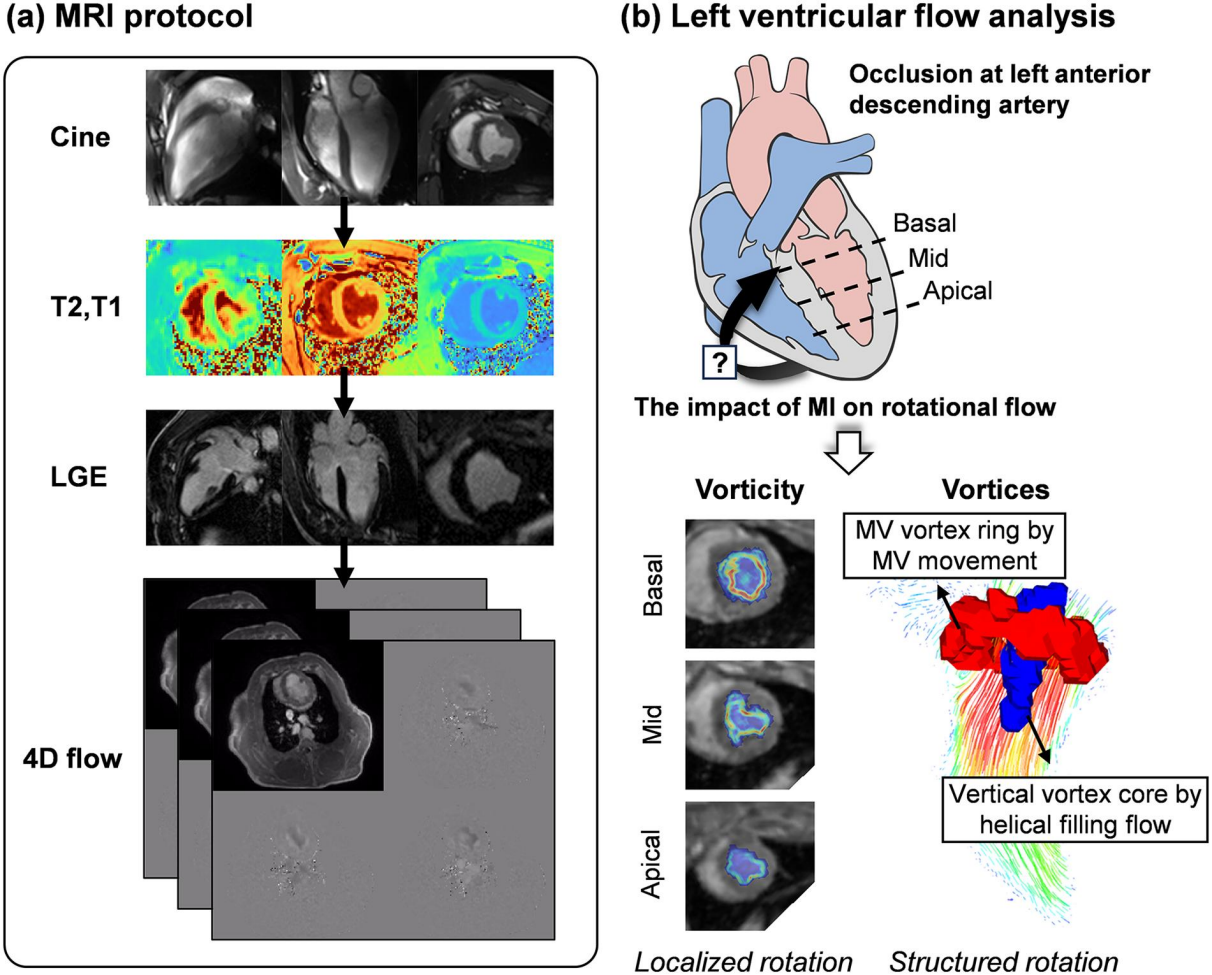
Overview of the study. **(a)** The MRI protocol comprised of cine imaging, native T1 and T2 mapping, late-gadolinium-enhancement (LGE) imaging, and 4D flow MRI over an 11-week follow-up period. **(b)** This study aimed to investigate whether MI at the left anterior descending artery could affect intracardiac rotational flow. Vorticity (local rotational flow) was quantified at the basal, mid, and apical planes, and vortex formation (structured coherent rotational flow) resulting from helical filling flow was evaluated. MI, myocardial infarction; MV, mitral valve. Heart diagram by Patrick J. Lynch (medical illustrator), licensed under CC BY 2.5.

All cardiac MRI (CMR) examinations were conducted using a commercial 3-T MRI scanner (Skyra, Siemens AG, Munich, Germany). 4D flow MRI data for pig 1 at 3W was excluded due to low image quality. The CMR protocols included cine and LGE imaging, native T1 and T2 mapping, and 4D flow acquisition (Figure 1a). ECG sensor was attached to a porcine model for cardiac MRI. LGE, T1 and T2 images were taken in a short-axis orientation, covering the complete LV after the administration of a gadolinium-based contrast agent. LGE images were acquired 20–30 min after contrast administration, and the extent of enhancement was quantified as the percentage of enhanced myocardial volume relative to total myocardial volume. T2 mapping was implemented after a protocol update and was therefore acquired only for Pig 1. MI was evaluated using LGE imaging and T1 and T2 mapping. For a detailed assessment of regional tissue characteristics, the LV myocardium was divided by following the American Heart Association (AHA)'s 16 segment guideline (22). The region with more than 25% of LGE was defined as infarcted based on the previous literature (23), while the others were defined as adjacent and remote regions based on their proximity to the infarcted region. LV global function was analyzed using a commercial software (CVI42, version 5.13.10, Circle Cardiovascular Imaging, Canada) by a clinician (Y.A.) with 9 years of CMR experiences. Global function and strain changes included end-diastolic volume (EDV) indexed to body surface area (BSA) [mL/m^2^], end-systolic volume (ESV) indexed to BSA [mL/m^2^], stroke volume (SV) indexed to BSA [mL/m^2^], cardiac output indexed to BSA (CI, Cardiac index) [L/min/m^2^], ejection fraction (EF) [%], myocardial mass indexed to BSA at end-diastole [g/m^2^], wall thickness at end-diastole [mm], global radial strain (GRS) [%], global circumferential strain (GCS) [%] and global longitudinal strain (GLS) [%]. 4D flow MRI images were acquired by a free-breathing sequence with retrospective ECG-gating and respiratory motion compensation. MRI acquisition parameters were as follows: For short-axis (SA) and long-axis (LAX) cine imaging: echo time (TE) = 1.45 ms (SA) and 1.55 ms (LAX), repetition time (TR) = 39.48 ms (SA) and 42.12 ms (LAX), flip angle = 36°–44°, voxel size = 1.56 × 1.56 × 10.8 mm^3^ (SA) and 1.25 × 1.25 × 8 mm^3^ (LAX). For T1 imaging: TE = 1.12 ms, TR = 281.64–316.48 ms, flip angle = 35°, voxel size = 1.41 × 1.41 × 12–19.8 mm^3^. For T2 imaging: TE = 1.32 ms, TR = 207.39–235.74 ms, flip angle = 12°, voxel size = 1.88 × 1.88 × 16.8–19.2 mm^3^. For LGE imaging: TE = 1.57 ms, TR = 452.4–770 ms, flip angle = 20°, voxel size = 1.37 × 1.37 × 12–21.6 mm^3^, with imaging performed 10 min post-contrast injection. For 4D flow MRI, TE = 2.69 ms, TR = 41.92 and 62.88 ms, flip angle = 15°, VENC = 150 cm/s and voxel size = 2.0 × 2.0 × 2.0 mm^3^ with 25 phases per cardiac cycle. Extracellular volume (ECV) was determined using pre- and post-contrast T1 values of blood and myocardium, following established literature methods (24).

### Intracardiac hemodynamic analysis

Intracardiac blood flow was obtained using magnitude and phase images acquired from 4D flow MRI technique. MR images were corrected for velocity anti-aliasing, noise filtering, and eddy current correction to enhance the accuracy of velocity quantification (25). 3D phase contrast (PC) magnetic resonance angiography images were further generated from 4D flow MRI images. After the MI introduction, significantly reduced flow signals were observed. Thus, the LV between fluid domain and non-fluid domain was manually segmented for each cardiac phase based on both PC MRA and magnitude images for LV flow analysis using an ITK-SNAP software (v.3.8.0, University of Utah, Salt Lake City, UT, USA). Flow rates were obtained at the basal plane, and utilized to estimate E-wave, A-wave, and E/A ratios.

Here, two rotational flow parameters were estimated as vorticity and vortex core. Vorticity, a local spinning of fluid particles, was quantified voxel-wise based on the mathematical definition of the curl of the velocity field. Vorticity contours were obtained at the basal, mid, and apical planes, which correspond to the locations of the LGE images (Representative contours at basal plane were shown in Supplementary Figure S1). The vorticity distribution within the LV blood pool was also divided according to a scheme analogous to the AHA's 16 myocardial segment guideline (Supplementary Figure S2). The infarct region was defined based on LGE. Then, vorticity values within the infarct, adjacent, and remote regions were spatially and temporally averaged from early diastole (phase 13) to end diastole (phase 25).

The vortex core, which is a collection of vortical flows rotating together through a common axis, was visualized using a Lambda2 method (26). In short, Lambda2 method uses the velocity gradient to obtain an eigenvalue, $\lambda_{2}$, which identifies regions of rotational flow by excluding the effects of the irrotational flow. The vortices can be identified by setting appropriate negative $\lambda_{2}$ value (See Supplementary Figure S3a), and qualitatively visualized using iso-surfaces of $\lambda_{2}$. Two distinctive vortices could be observed: (1) mitral valve (MV) vortex ring by the movement of MV and (2) vertical vortex core (VVC) by helical filling flow (Figure 1a).

In the present study, we assessed progressive changes in diastolic helical filling flow by quantifying the orientation and angle of VVC, with the goal of providing mechanistic insight into LV remodeling rather than focusing on MV dynamics. The VVC centerline was defined by generating a line passing through the center of the extracted VVC (Supplementary Figure S3b). The anatomical centerline was subsequently defined on magnitude MR images by connecting the centers of the basal and apical planes (Supplementary Figure S3c), providing a stable geometric reference that would be less sensitive to regional deformation or displacement at the mid-ventricular level where MI occurred.

The orientation of the VVC was defined by the point at which its centerline crosses the mid-plane (conceptually illustrated as a dot on the LV blood pool; see Supplementary Figure S2b for reference), while the angle between the VVC and the anatomical centerlines was calculated as a value in radians in angular plots. Both orientation and angle were plotted in a radial direction, where the maximum variation of angle was set to π/6 for clear visualization of tendencies. The analysis was primarily conducted at early diastolic phase (E-wave), where the VVC is strongly formed. However, when the VVC was not clearly identified at E-wave due to abnormal diastolic inflow patterns, a later diastolic phase where VVC was clearly formed was utilized for the assessment. As a proof-of-concept application, the analysis was performed by an engineer (S.P.) with 3 years of experience in 4D flow MRI.

### Statistical analysis

For each cardiac parameter, temporal trajectories within each animal were summarized. Overall temporal trends were quantified using the Theil–Sen estimator, a robust non-parametric method that estimates the median slope across all pairwise time points. Uncertainty in the estimated slopes was assessed using a residual bootstrap approach with 1,000 resamples, in which residuals from the fitted trend were resampled, added back to the fitted trajectory, and the slope re-estimated for each bootstrap sample. The resulting bootstrap distributions were used to derive 95% confidence intervals (CI).

Temporal changes are therefore reported as slope estimates with corresponding 95% CIs. A positive slope indicates an increasing trend, whereas a negative slope indicates a decreasing trend. When the 95% CI lies predominantly on one side of zero, including cases where the interval marginally crosses zero with a small opposite sign, the trend may be interpreted as predominantly unidirectional. When the 95% CI spans both positive and negative values substantially, no clear directional trend is inferred, potentially reflecting non-monotonic or transient changes over the study timeline.

In addition, correlation analyses were performed to examine cross-sectional associations among MRI- and 4D flow MRI-derived parameters across all time points. Pearson correlation coefficients were used when normality assumptions were met, whereas Spearman rank correlations were applied for non-parametric distributions.

## Results

### MI-induced LV remodeling

**Figure 2:**
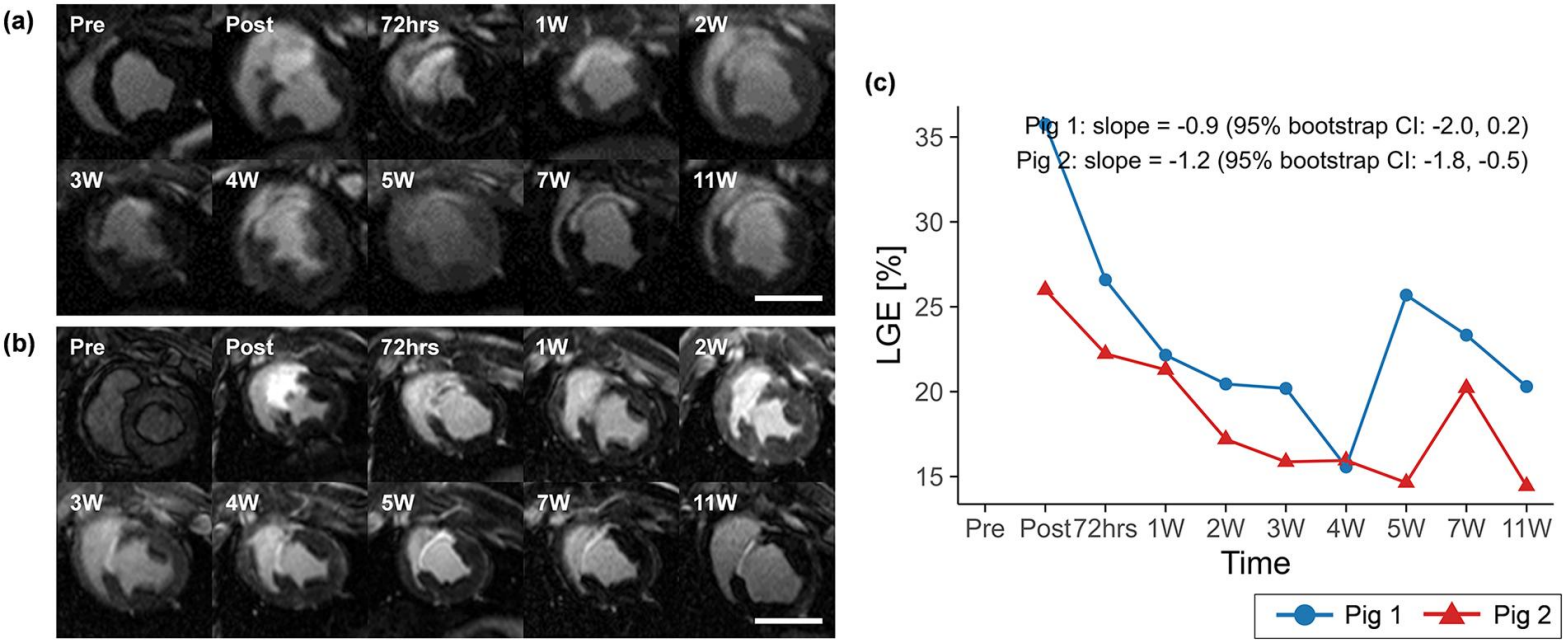
Mid-ventricular LGE images for **(a)** pig 1 and **(b)** pig 2, and **(c)** temporal changes in LGE over the study period. LGE extent is shown descriptively across time points to illustrate longitudinal patterns within each animal, with temporal trends summarized by Theil–Sen slope estimates and 95% residual bootstrap confidence intervals. Overall, LGE tended to decrease over time, with a transient increase at 5 weeks in pig 1 and at 7 weeks in pig 2. Scale bars indicate 50 mm. LGE, late gadolinium enhancement.

Territorial transmural infarction in the mid-ventricular anteroseptal region was identified by LGE imaging in both porcine models (Figures 2a,b). Representative cine magnitude images at the mid-ventricular plane for pig 1 are provided in Supplementary Video S1. Following acute occlusion of the mid LAD, the infarcted region measured by LGE decreased from 35.8% at post-MI to 20.3% at 11 weeks in pig 1, with a slope of −0.9 (95% CI: −2.0 to 0.2), and from 26.0% to 14.4% in pig 2, with a slope of −1.2 (95% CI: −1.8 to −0.5) (Figure 2c and Table 1).

**Table 1:** Temporal variations of LGE, native T1 and T2 mapping, and ECV for pig 1 and 2.

Variable	Pig	Region	Pre	Post	72hrs	1W	2W	3W	4W	5W	7W	11W	Slope
LGE [%]	Pig 1	Infarct		35.8	26.6	22.2	20.5	20.2	15.6	25.7	23.3	20.3	−0.9 (−2.0‒0.2)
	Pig 2	Infarct		26.0	22.2	21.3	17.2	15.9	16.0	14.6	20.2	14.4	−1.2 (−1.8‒−0.5)
Native T1 relaxation time [ms]	Pig 1	Infarct	1,079	1,153	1,326	1,319	1,323	1,183	1,366	1,277	1,395	1,310	14.4 (0.0‒29.2)
		Adjacent	1,086	1,054	1,088	1,072	1,110	1,024	1,087	1,002	1,092	1,072	−0.3 (−7.1‒5.4)
		Remote	1,090	819	912	941	923	892	866	944	887	929	−1.2 (−12.1‒11.1)
	Pig 2	Infarct	919	1,089	1,098	965	997	956	1,141	1,114	1,427	1,083	15.9 (−4.2‒38.6)
		Adjacent	935	960	960	975	907	867	862	962	1,022	846	−3.2 (−12.1‒6.7)
		Remote	972	1,000	903	980	861	847	670	867	952	890	−10.0 (−28.1‒5.0)
ECV [%]	Pig 1	Infarct	23	63	61	69	66	63	64	66	57	64	0.3 (−0.9‒1.2)
		Adjacent	22	28	25	25	25	27	28	30	20	26	0.1 (−0.4‒0.7)
		Remote	26	26	27	28	27	28	29	25	23	25	−0.1 (−0.4‒0.2)
	Pig 2	Infarct	29	34	48	46	51	48	63	44	42	49	1.4 (−0.2‒3.0)
		Adjacent	24	19	19	18	17	21	27	21	26	23	0.4 (−0.1‒1.0)
		Remote	24	31	22	24	18	13	30	26	25	17	−0.5 (−1.6‒0.6)
T2 relaxation time [ms]	Pig 1	Infarct	46	69	86	97	76	77	67	66	77	62	−0.6 (−3.2‒1.8)
		Adjacent	43	47	57	46	42	46	42	43	46	43	−0.2 (−0.7‒0.5)
		Remote	41	41	45	43	39	46	39	41	46	31	−0.5 (−1.2‒0.4)

Slopes are expressed as Theil–Sen estimates with 95% confidence intervals derived from residual bootstrap resampling.

**Figure 3:**
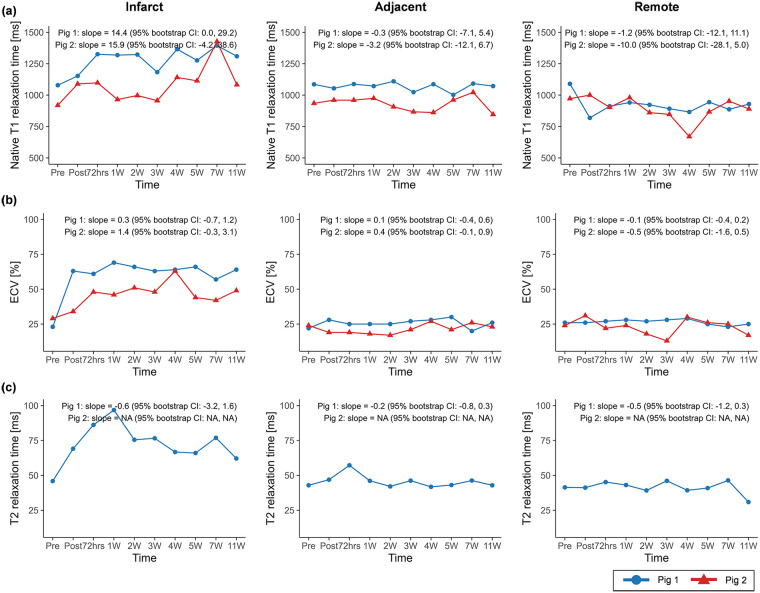
Temporal changes in **(a)** native T1 relaxation time, **(b)** extracellular volume (ECV), and **(c)** T2 relaxation time across infarct, adjacent, and remote regions. Temporal trajectories are summarized descriptively, with overall trends characterized using Theil–Sen slope estimates and 95% residual bootstrap confidence intervals. Native T1 relaxation time exhibited clearer increasing tendencies compared with ECV and T2 relaxation time across regions. ECV, extracellular volume.

The native T1 relaxation time within the infarct region showed increasing trends over the study timeline in both pig 1 (slope = 14.4, 95% CI: 0.0 to 29.2) and pig 2 (slope = 15.9, 95% CI: −4.2 to 38.6) (Figure 3), whereas ECV and T2 relaxation time exhibited less consistent temporal patterns.

**Figure 4:**
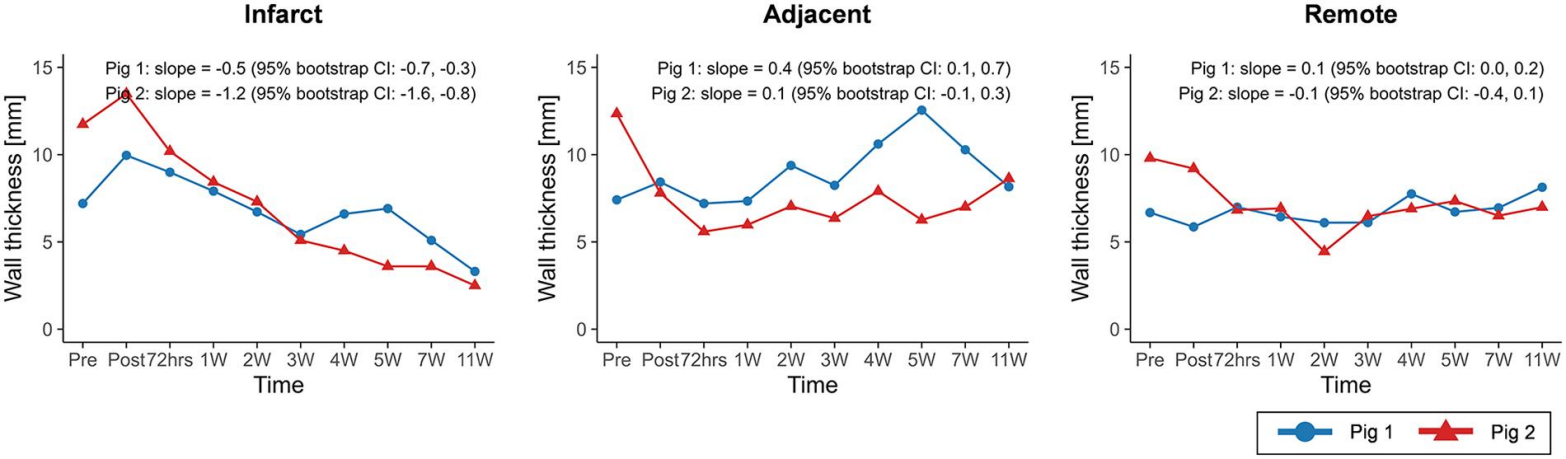
Temporal changes in wall thickness across infarct, adjacent, and remote regions. Temporal trajectories are summarized descriptively, with overall trends characterized using Theil–Sen slope estimates and 95% residual bootstrap confidence intervals. Wall thickness in the infarct region exhibited a consistent decreasing trend over time in both animals.

**Figure 5:**
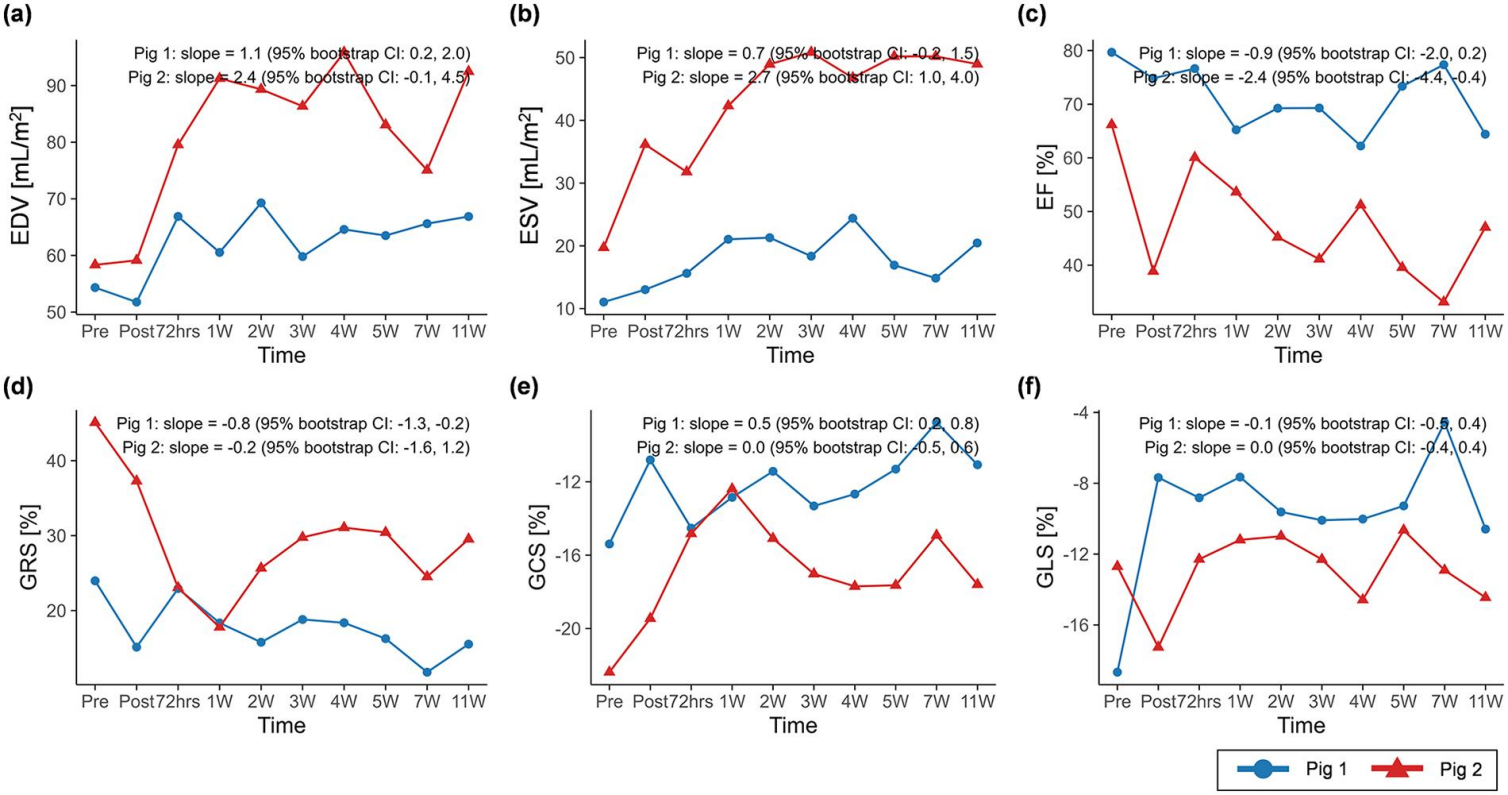
Temporal changes in **(a)** EDV, **(b)** ESV, **(c)** EF, **(d)** GRS, **(e)** GCS, and **(f)** GLS over the study period. Temporal trajectories are summarized descriptively, with overall trends characterized using Theil–Sen slope estimates and 95% residual bootstrap confidence intervals. EDV and ESV exhibited clearer increasing trends over time than EF and global peak strains. BSA, body surface area; EDV, end-diastolic volume indexed to BSA; ESV, end-systolic volume indexed to BSA; EF, ejection fraction; GRS, global radial strain; GCS, global circumferential strain; GLS, global longitudinal strain.

The CMR results of global function and strain changes are summarized in Table 2. Wall thickness in the infarct region exhibited decreasing trends over time in both porcine models (slope = −0.5, 95% CI: −0.7 to −0.3 in pig 1; slope = −1.2, 95% CI: −1.6 to −0.8 in pig 2) (Figure 4). In both animals, EDV (slope = 1.1, 95% CI: 0.2 to 2.0 in pig 1; slope = 2.4, 95% CI: −0.1 to 4.5 in pig 2) and ESV (slope = 0.7, 95% CI: −0.2 to 1.5 in pig 1; slope = 2.7, 95% CI: 1.0 to 4.0 in pig 2) showed progressive increases, whereas EF demonstrated decreasing trends (slope = −0.9, 95% CI: −2.0 to 0.2 in pig 1; slope = −2.4, 95% CI: −4.4 to −0.4 in pig 2) (Figure 5).

**Table 2:** Temporal variations of left ventricular global functions and strains for pig 1 and 2.

Variable	Pig	Region	Pre	Post	72hrs	1W	2W	3W	4W	5W	7W	11W	Slope
EDV [mL/m^2^]	Pig 1		54.3	51.8	66.9	60.5	69.3	59.8	64.6	63.5	65.6	66.9	1.1 (0.2‒2.0)
	Pig 2		58.3	59.1	79.6	91.3	89.3	86.4	95.9	83.1	75.1	92.5	2.4 (−0.1‒4.5)
ESV [mL/m^2^]	Pig 1		11.1	13.0	15.6	21.1	21.3	18.4	24.4	16.9	14.9	20.5	0.7 (−0.2‒1.5)
	Pig 2		19.7	36.2	31.8	42.3	48.9	50.8	46.8	50.2	50.2	49.0	2.7 (1.0‒4.0)
SV [mL/m^2^]	Pig 1		43.3	38.7	51.3	39.5	48.0	41.4	40.2	46.6	50.8	46.4	0.6 (−0.3‒1.4)
	Pig 2		38.6	23.0	47.8	48.9	40.4	35.5	49.1	32.9	24.9	45.3	−0.2 (−2.2‒1.7)
CI [L/min/m^2^]	Pig 1		3.5	2.9	4.0	3.4	4.0	3.7	3.4	4.0	3.8	3.6	0.0 (−0.1‒0.1)
	Pig 2		3.9	2.3	4.8	5.9	4.6	4.0	4.0	3.4	2.9	4.4	−0.1 (−0.3‒0.1)
EF [%]	Pig 1		79.7	74.8	76.6	65.2	69.3	69.3	62.2	73.3	77.4	64.4	−0.9 (−2.0‒0.2)
	Pig 2		66.2	38.9	60.1	53.6	45.2	41.1	51.2	39.6	33.1	47.1	−2.4 (−4.4‒−0.4)
Myocardial mass [g/m^2^]	Pig 1		56.3	55.5	61.0	57.1	55.3	59.6	73.6	58.7	58.6	61.8	0.5 (0.0‒1.0)
	Pig 2		59.3	60.3	65.2	62.6	57.8	65.5	53.6	56.2	55.1	69.0	−0.5 (−1.3‒0.6)
Wall thickness [mm]	Pig 1	Infarct	7.2	10.0	9.0	7.9	6.7	5.4	6.6	6.9	5.1	3.3	−0.5 (−0.7‒−0.3)
		Adjacent	7.4	8.4	7.2	7.3	9.4	8.2	10.6	12.6	10.3	8.2	0.4 (0.1‒0.7)
		Remote	6.7	5.9	7.0	6.4	6.1	6.1	7.8	6.7	7.0	8.1	0.1 (0.0‒0.2)
	Pig 2	Infarct	11.7	13.5	10.2	8.4	7.3	5.1	4.5	3.6	3.6	2.5	−1.2 (−1.6‒−0.8)
		Adjacent	12.4	7.8	5.6	6.0	7.0	6.4	7.9	6.3	7.0	8.6	0.1 (−0.1‒0.3)
		Remote	9.8	9.2	6.8	6.9	4.5	6.5	6.9	7.3	6.5	7.0	−0.1 (−0.4‒0.1)
GRS [%]	Pig 1		24.0	15.1	22.9	18.4	15.8	18.8	18.4	16.3	11.8	15.5	−0.8 (−1.3‒−0.2)
	Pig 2		45.1	37.3	23.1	17.8	25.7	29.8	31.1	30.4	24.5	29.5	−0.2 (−1.6‒1.2)
GCS [%]	Pig 1		−15.4	−10.8	−14.5	−12.9	−11.4	−13.3	−12.7	−11.3	−8.8	−11.1	0.5 (0.2‒0.8)
	Pig 2		−22.4	−19.5	−14.8	−12.4	−15.1	−17.0	−17.7	−17.6	−14.9	−17.6	0.0 (−0.5‒0.6)
GLS [%]	Pig 1		−18.7	−7.7	−8.8	−7.7	−9.6	−10.1	−10.0	−9.3	−4.6	−10.6	−0.1 (−0.5‒0.4)
	Pig 2		−12.7	−17.3	−12.3	−11.2	−11.0	−12.3	−14.6	−10.7	−12.9	−14.5	0.0 (−0.4‒0.4)

Slopes are expressed as Theil–Sen estimates with 95% confidence intervals derived from residual bootstrap resampling.

Wall thickness was analyzed by infarct, adjacent, and remote regions, whereas global function and strain were assessed without regional classification.

### MI-induced rotational flow changes

**Figure 6:**
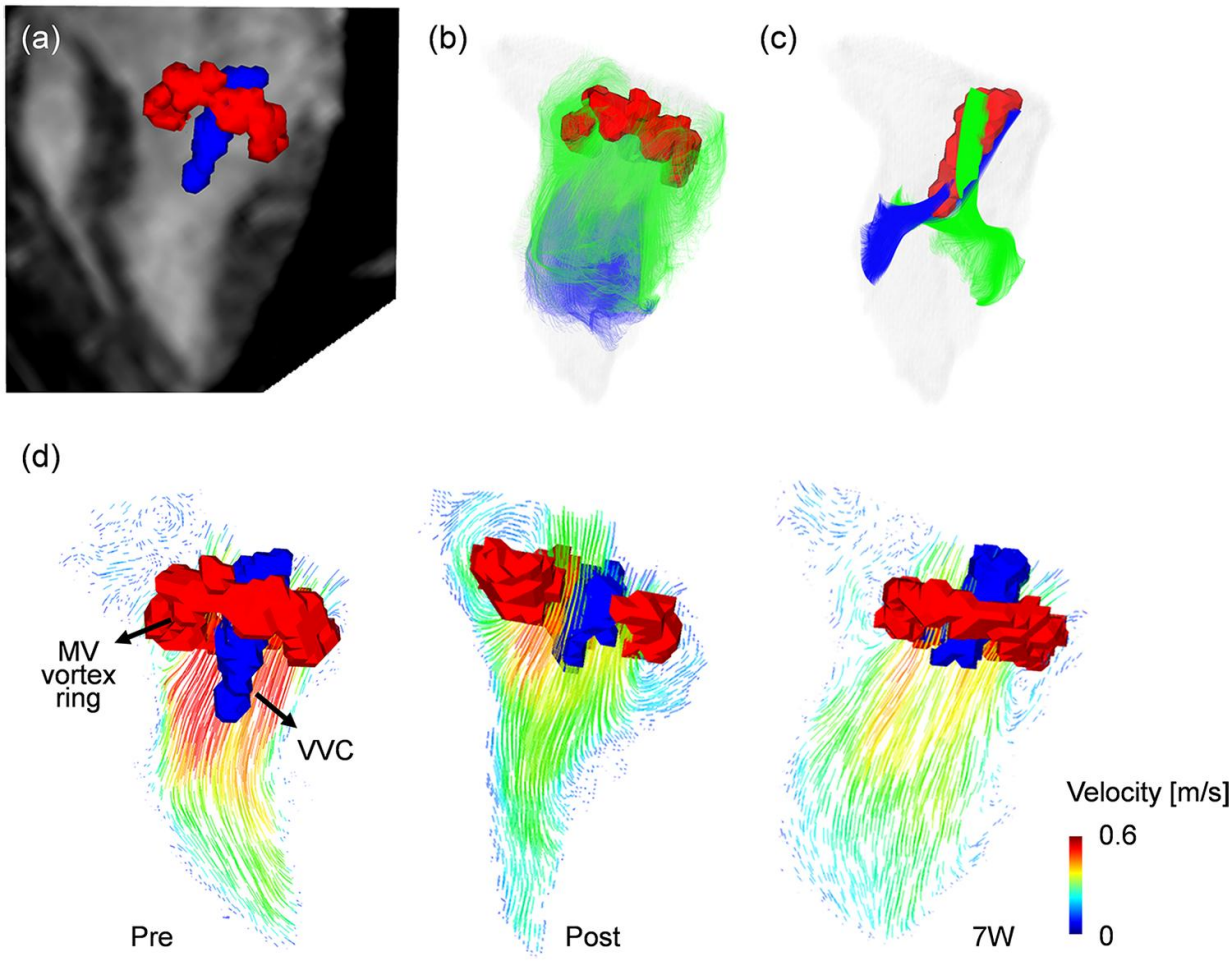
Visualization of vortex cores by using a Lambda2 method. **(a)** Two distinctive vortex cores of mitral valve (MV, red color) vortex ring and vertical vortex core (VVC, blue color) with 3-chamber MR image at the early diastolic phase (E-wave). **(b)** MV vortex ring visualized by particle trace analysis, with trace particles seeded at the MV level showing rotational motion along the LV wall and passage through the MV annulus during valve opening. **(c)** VVC visualized by particle trace analysis, with trace particles exclusively seeded near the MV center at the VVC formation region exhibiting coherent rotational motion that forms a three-dimensional helical flow pattern within the central LV cavity. **(d)** Representative variations in the MV vortex ring (red) and VVC (blue), together with the associated two-dimensional velocity vector fields, illustrating differences in inflow pattern and intensity across follow-up time points.

**Figure 7:**
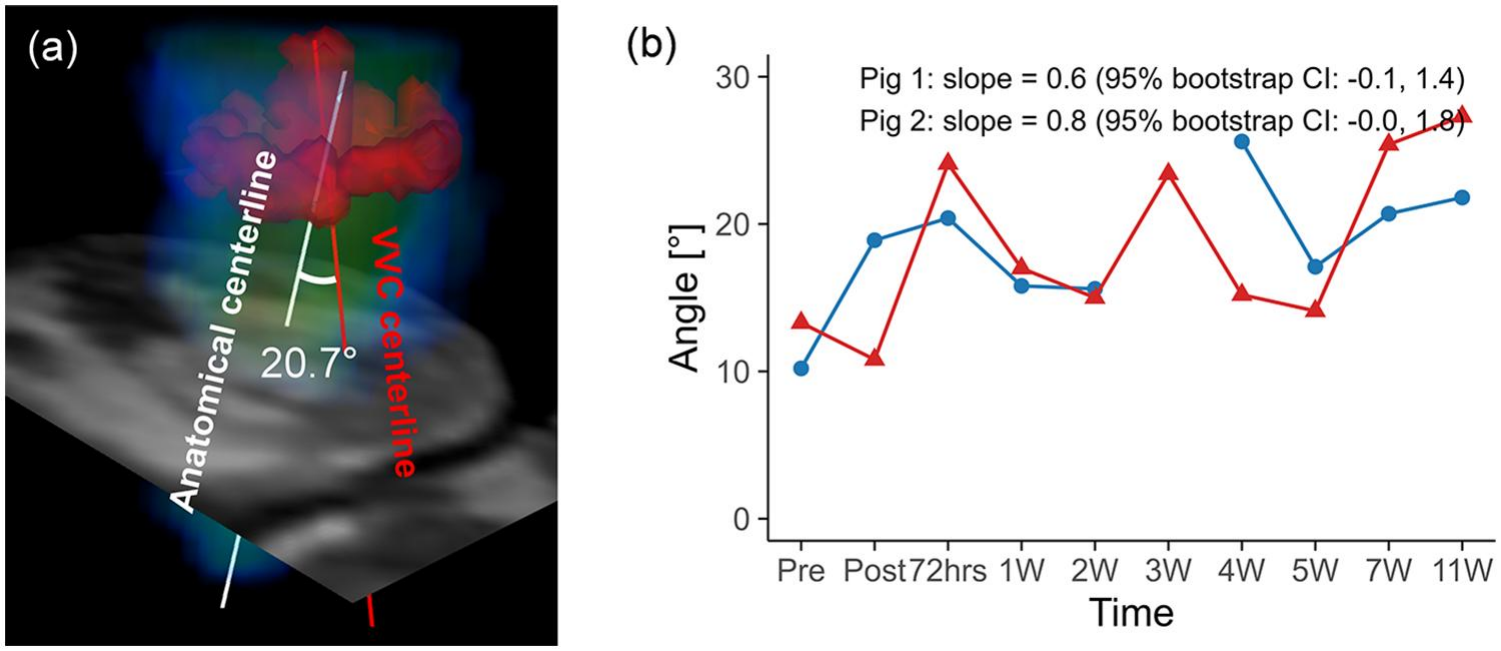
**(a)** Schematic representation of angle quantification for the vertical vortex core (VVC), with the VVC centerline shown in red and the anatomical centerline shown in white. **(b)** Temporal changes in the angle over the study period for pig 1 and pig 2. Temporal trajectories are summarized descriptively, with overall trends characterized using Theil–Sen slope estimates and 95% residual bootstrap confidence intervals. The angle exhibited an overall increasing tendency over time in both animals.

Representative 4D flow MRI visualizations, including MR magnitude images, particle traces, velocity fields, and vortex structures, are shown in Figure 6, where helical flow was observed only in particle traces seeded at the VVC (Supplementary Video S2). The schematic for angle quantification is shown in Figure 7a. Progressive reductions in myocardial wall motion, particularly within regions affected by MI, were observed at pre-MI, 4W, and 11W with flow visualizations (Supplementary Video S3). The VVC angle showed increasing temporal trends in both animals (slope = 0.6, 95% CI: −0.1 to 1.4 in pig 1; slope = 0.8, 95% CI: 0.0 to 1.8 in pig 2) (Figure 7b). However, temporal changes in regional vorticity showed no clear temporal tendencies (Supplementary Figure S4). The E/A flow ratio, average vorticity at the infarct, adjacent, and remote regions, and the angle between the anatomical center and the VVC center are summarized in Table 3.

**Table 3:** Temporal variations of the angle between the vertical vortex core and the anatomical center, the mitral valve E/A flow ratio, and the vorticity in the basal, mid, and apical planes for pig 1 and 2.

Variable	Pig	Region	Pre	Post	72hrs	1W	2W	3W	4W	5W	7W	11W	Slope
Angle [°]	Pig 1		10.6	18.9	20.4	15.8	15.6	n/a	25.6	17.1	20.7	21.8	0.6 (−0.1‒1.4)
	Pig 2		13.3	10.8	24.1	17.0	15.0	23.4	15.2	14.1	25.4	27.3	0.8 (0.0‒1.8)
E/A flow ratio [-]	Pig 1		1.7	0.9	1.4	1.1	1.3	n/a	2.6	1.8	1.8	1.3	0.0 (0.0‒0.1)
	Pig 2		0.9	2.0	1.7	0.5	0.1	1.3	0.1	0.1	0.1	1.2	−0.1 (−0.2‒0.0)
Vorticity in the basal plane [1/s]	Pig 1	Infarct	39.4	33.2	32.4	36.9	38.3	n/a	39.0	34.9	34.9	35.6	−0.0 (−0.5‒0.4)
		Adjacent	38.9	29.4	32.6	34.1	35.2	n/a	38.5	32.9	31.8	31.5	−0.2 (−0.7‒0.4)
		Remote	38.6	33.6	36.0	37.0	37.4	n/a	39.9	41.1	35.9	35.6	0.3 (−0.2‒0.7)
	Pig 2	Infarct	34.8	30.3	29.8	30.2	35.9	30.9	35.1	30.7	32.3	27.4	−0.1 (−0.6‒0.4)
		Adjacent	30.8	31.3	31.3	26.6	31.7	26.7	30.9	28.5	30.8	26.5	−0.1 (−0.5‒0.3)
		Remote	30.6	28.0	28.6	28.3	31.7	30.6	29.9	28.0	29.1	27.7	−0.1 (−0.3‒0.2)
Vorticity in the mid plane [1/s]	Pig 1	Infarct	30.1	26.2	26.3	18.2	22.6	n/a	29.6	25.9	23.1	24.4	−0.2 (−0.9‒0.4)
		Adjacent	29.2	27.8	29.8	24.3	24.3	n/a	31.0	31.1	27.3	26.2	0.0 (−0.5‒0.5)
		Remote	27.9	24.9	23.4	23.0	25.1	n/a	33.0	32.0	26.5	28.3	0.3 (−0.2‒0.9)
	Pig 2	Infarct	11.9	16.0	21.4	12.3	13.7	11.4	13.3	12.3	12.3	13.8	0.0 (−0.4‒0.3)
		Adjacent	17.0	19.1	23.7	13.8	16.6	14.9	17.4	15.9	15.1	14.6	−0.3 (−0.7‒0.1)
		Remote	20.4	18.8	19.4	16.3	21.4	16.6	22.7	18.3	19.6	14.5	−0.3 (−0.7‒0.2)
Vorticity in the apical plane [1/s]	Pig 1	Infarct	16.0	21.8	18.1	14.6	13.2	n/a	17.0	15.6	14.0	15.8	−0.3 (−0.7‒0.2)
		Adjacent	17.6	20.4	19.1	12.9	13.2	n/a	17.7	17.8	15.8	14.4	−0.4 (−0.8‒0.0)
		Remote	19.7	18.7	18.6	12.4	14.6	n/a	18.8	18.7	16.0	16.6	−0.2 (−0.7‒0.1)
	Pig 2	Infarct	9.6	16.5	22.7	10.5	9.0	9.4	9.9	9.6	10.7	8.2	−0.2 (−0.5‒0.3)
		Adjacent	10.5	13.1	16.8	9.2	10.6	9.3	10.8	11.1	10.2	7.6	−0.3 (−0.6‒0.1)
		Remote	11.6	13.0	15.9	8.9	13.4	10.2	13.0	12.2	10.5	7.3	−0.4 (−0.8‒0.0)

n/a indicates not applicable due to low image quality. Slopes are expressed as Theil–Sen estimates with 95% confidence intervals derived from residual bootstrap resampling

Angle and E/A flow ratio were assessed without regional classification.

We also observed differences in baseline VVC orientation between the two animals: in pig 1, the pre-MI VVC was oriented toward the inferoseptal region, whereas in pig 2, the pre-MI VVC was oriented toward the anterolateral region (Supplementary Figure S5). As remodeling progressed following MI, the VVC orientation appeared to shift clockwise relative to the pre-MI state in both pigs.

Correlations among tissue characteristics, global function, global strain, and 4D flow parameters were evaluated across all time points, capturing cross-sectional associations between variables (Supplementary Figures S6 and S7). While vorticity did not show clear temporal changes and remained relatively preserved over time (Table 3), regional associations with infarct size were observed. Specifically, LGE extent was negatively correlated with basal vorticity at the infarct region in pig 1 (r = −0.81) and positively correlated with apical vorticity at the infarct region in pig 2 (rho = 0.85).

## Discussion

This study demonstrates that LV remodeling progression can be accompanied by distinct intracardiac flow patterns. The key findings simultaneously observed in both animals are as follows: (1) Myocardial tissue characteristics showed differential temporal behavior, with LGE extent and native T1 relaxation time exhibiting clearer longitudinal changes than ECV and T2 relaxation time. (2) Structural remodeling measures, including infarct-region wall thickness, EDV, and ESV, exhibited more pronounced temporal changes than conventional global functional metrics such as EF and GLS. (3) The angle of helical filling flow progressively increased, while regional vorticity was preserved.

Previous studies have characterized post-MI remodeling through longitudinal cardiac MRI, which includes cine, LGE imaging, or native T1/T2 mapping, in porcine models (27, 28) or large cohorts (29, 30). However, no prior work has simultaneously examined LV remodeling and detailed intracardiac flow features over time. Thus, the present study underscores the potential of 4D flow-based rotational flow analysis to provide mechanistic insight into LV adaptation in MI porcine models.

### Traditional MI characterization and LV remodeling

LV remodeling after MI has been well established using LGE imaging, native T1, T2 mapping, and ECV (31–33). LGE typically shows the largest infarct extent immediately after MI, followed by a gradual decrease over the first 35 days, after which values tend to stabilize (34). This pattern was consistent with our observations, with a gradual decrease up to 4W in pig 1 and 5W in pig 2. Notably, a secondary increase in LGE was observed at 5W in pig 1 and 7W in pig 2. This may be related to outliers reported at Day 35 and Day 180 in prior work (34), suggesting that transient fluctuations in LGE may occur depending on the imaging time point.

Given that normal myocardial ECV is approximately 25% (35) and native T1 values near 1,050 ms in healthy myocardium (36), the baseline measurements in our porcine models indicate extracellular tissue properties comparable to those in humans. Following acute MI, myocardial reperfusion increases myocardial water content by ∼28% (37), which leads to elevations in T1 and T2 relaxation times (38, 39). As edema resolves, T1 and T2 gradually decrease over the following weeks to months, which helps distinguish acute from chronic MI, while ECV typically decreases by around 7 weeks post-MI (40).

In contrast, because the present study sampled densely between baseline and 11W, we observed a different temporal pattern: native T1, T2 and ECV values in the infarct region initially rose within the first week, followed by a transient decline, and then demonstrated secondary increases during the early chronic phase. Thus, these metrics should be interpreted with caution, because applying the previously reported monotonic decrease from acute to chronic MI may overlook the temporal fluctuations that occur throughout the remodeling process. Accordingly, interpretation of tissue parameter changes across MI stages may benefit from integration with complementary structural or functional markers, rather than relying solely on their temporal behavior.

### Global functions and strains

Decreases in wall thickness were observed in the infarct region in both pigs. In pig 1, wall thickness increased in the adjacent and remote regions, which may reflect compensatory hypertrophy during scar maturation (41, 42). Enlargement of EDV and ESV is a well-recognized feature of LV remodeling following MI (43, 44), and the increasing trends observed for EDV and ESV in both pigs are consistent with this remodeling pattern.

Poor recovery of EF is a known predictor of adverse cardiac events and sudden death (41). In the present study, EF did not recover and instead exhibited declining trends with temporal fluctuations, which may be consistent with a less favorable remodeling trajectory in both models. GLS has also been proposed as a marker of LV remodeling, particularly when GLS exceeds −15%, reflecting concomitant ventricular dilatation (42). This is consistent with our findings, where GLS mostly remained >−15% in both pigs, which potentially induced EDV and ESV dilatation.

In pig 2, the decline in EF was more pronounced, and the rates of EDV and ESV dilatation were steeper compared with pig 1, suggesting a more rapid progression of LV remodeling. However, GLS values remained close to the pre-MI level, which was already above the −15% threshold. In contrast, GRS and GCS exhibited larger temporal variations and showed close associations with wall thickness in the adjacent and remote regions across all time points (Supplementary Figure S7). Given that pig 2 demonstrated limited compensatory hypertrophy compared with pig 1, these findings suggest that LV remodeling and functional adaptation may be regionally heterogeneous. Baseline differences and temporal variation in LV functional parameters should be considered when evaluating the severity and potential prognostic implications of LV remodeling.

### Intracardiac rotational flow

Ventricular diastolic dysfunction in LV remodeling has been associated with reduced vorticity in conditions such as chronic obstructive pulmonary disease and non-ischemic dilated or hypertrophic cardiomyopathies (45, 46). This reduced vorticity reflects diminished rotational flow energetics and impaired kinetic energy transfer. In MI, decreased vorticity has also been reported due to ventricular dilation and regional wall motion abnormalities, whereas the presence of left ventricular thrombus can elevate vorticity by altering the local velocity field and diastolic filling direction (18).

In the present study, despite hypocontractile deformation in the mid-anteroseptal infarct region (Supplementary Videos S1 and S2), regional vorticity values across basal, mid, and apical planes and infarct, adjacent, and remote regions did not show clear trends over time, indicating preserved rotational flow energetics. This observation contrasts with a prior in silico simulation study that explicitly modeled scar-induced myocardial stiffening through non-contractile wall motion and reported increased vorticity magnitude after MI (47), where intracardiac flow was primarily driven by prescribed wall motion and stroke volume magnitude without considering inflow orientation.

In contrast, our *in vivo* longitudinal data revealed a progressive clockwise shift in diastolic filling direction with increasing trends of the inflow angle, assessed by the VVC, in both pigs relative to their pre-MI baseline. While hypocontractile deformation alone might be expected to increase vorticity, concurrent changes in inflow orientation may redistribute rotational flow within the LV, thereby preserving overall vorticity despite impaired regional contractility. Supporting this interpretation, a prior clinical study showed that the orientation of the diastolic vortex ring differed significantly between MI patients with and without LV thrombus, despite no differences in vorticity magnitude (18). As such, these findings suggest that adaptation of diastolic inflow orientation, beyond wall motion abnormalities alone, may contribute to the preservation of rotational flow during post-MI remodeling.

### Inter-animal variability in 4D flow characteristics and mechanistic implications

A previous study reported an inverse association between infarct size and mean vorticity values (18). Consistent with this finding, pig 1 demonstrated a negative correlation between infarct size estimated by LGE and vorticity within the infarct region at the basal plane. In contrast, pig 2 did not exhibit this inverse relationship, but instead showed a positive association between infarct size and apical vorticity within the infarct region.

Differences in inflow orientation between the pigs, present even at baseline, are expected to influence regional vorticity characteristics, as the direction of incoming flow strongly shapes local flow structures, even when infarct location is similar. According to guidance from the British Society of Echocardiography (48), LV dilation may cause transmitral inflow to be directed postero-laterally due to tethering of the mitral leaflets. Consistent with this framework, the observed change in VVC orientation in pig 2, used here as a marker of inflow direction, from an anterior–anterolateral to an inferolateral direction may reflect the more pronounced LV dilation following MI.

In addition, the differences in regional vorticity may be further amplified by a markedly reduced E/A ratio in pig 2, indicating impaired early diastolic suction and a greater reliance on atrial contraction for ventricular filling. Such A-wave dominant filling may result in prolonged residence of inflowing blood and redistribution of flow momentum toward the apical region. In combination with altered inflow orientation, these dynamics may promote residual rotational flow deeper within the LV, contributing to increased apical vorticity with larger infarct size.

As such, these observations suggest that inflow characteristics, including orientation and filling dynamics, may underlie inter-pig differences in the associations between structural measures and flow-related parameters obtained from 4D flow MRI. Considering inflow-based stratification may therefore be informative when interpreting structure–flow relationships in post-MI remodeling.

### Limitations

This study is limited by the small sample size (*n* = 2), which precludes statistical inference and generalizability. Accordingly, the findings are interpreted in an exploratory and descriptive manner based on observed temporal trends. The absence of non-MI control pigs further limits causal interpretation. Nevertheless, the dense longitudinal follow-up from baseline to 11 weeks enables detailed characterization of post-MI LV remodeling that cannot be captured in cross-sectional studies. Histological validation was not performed, limiting direct confirmation of imaging-derived tissue and flow changes. Nevertheless, the longitudinal T1 and T2 trends observed in the present study are consistent with prior reports of post-MI remodeling.

Although the infarct location was similar between models, they exhibited distinct remodeling and flow responses, suggesting that variations in infarct size and severity may influence subsequent adaptation. Further studies with larger cohorts are needed to delineate the range and determinants of remodeling phenotypes. 4D flow MRI-derived parameters may be influenced by spatiotemporal resolution. Because imaging parameters were kept constant across all time points in the present study, the analysis emphasizes relative temporal trends across the study timeline rather than absolute values.

Manual segmentation may introduce observer-dependent variability, particularly for vorticity-related measures. However, vorticity remained relatively preserved in the present study. In contrast, the VVC angle showed consistent increasing trends in both pigs and is less sensitive to segmentation boundaries, thereby reducing the impact of segmentation-related bias. The helical filling flow assessed using the VVC may not translate directly to humans, as helical inflow patterns are more commonly observed in the right atrium than the LV (49). Nevertheless, the observed progressive shift in vortex orientation provides insight into adaptation of diastolic filling flow during remodeling. Future work will include larger patient cohorts to validate the relationship between diastolic flow organization and LV remodeling and to evaluate its potential utility as a prognostic biomarker for post-MI progression.

## Conclusion

We longitudinally tracked LV remodeling and rotational flow from baseline to 11 weeks in two porcine MI models. Although both models exhibited overall similar post-MI remodeling, they followed distinct structural and functional progression patterns with differences in the absolute magnitude of the measured parameters, highlighting individualized remodeling trajectories. Notably, intracardiac vorticity magnitude remained preserved, whereas the angle of the diastolic filling vortex increased in both models, suggesting its potential relevance to remodeling progression and warranting validation in larger cohorts.

## Data Availability

The raw data supporting the conclusions of this article will be made available by the authors, without undue reservation.
