# Supplementary material for: Longitudinal observation of left ventricular inflow reorientation with preserved vorticity after myocardial infarction in a porcine model
Source: Front Cardiovasc Med. 2026 Feb 6;13:1742432. doi: 10.3389/fcvm.2026.1742432 (PMC12920533; doi:10.3389/fcvm.2026.1742432)
Supplement: Supplementary file 4 [file Datasheet1.docx]

**
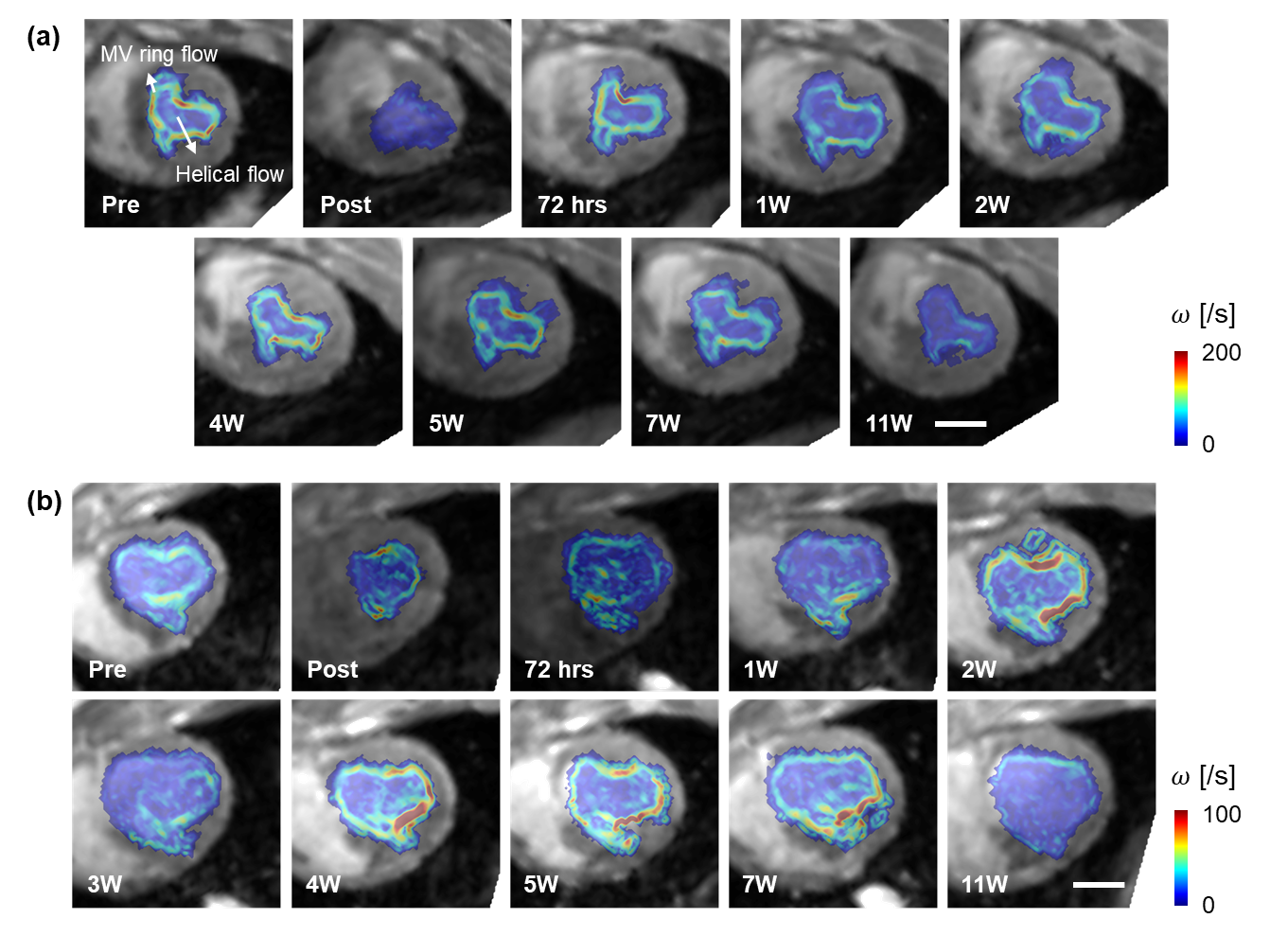
**

Supplementary Figure S1. Vorticity distributions during our study period in (a) pig 1 and (b) pig 2. Scale bars indicate 25 mm.


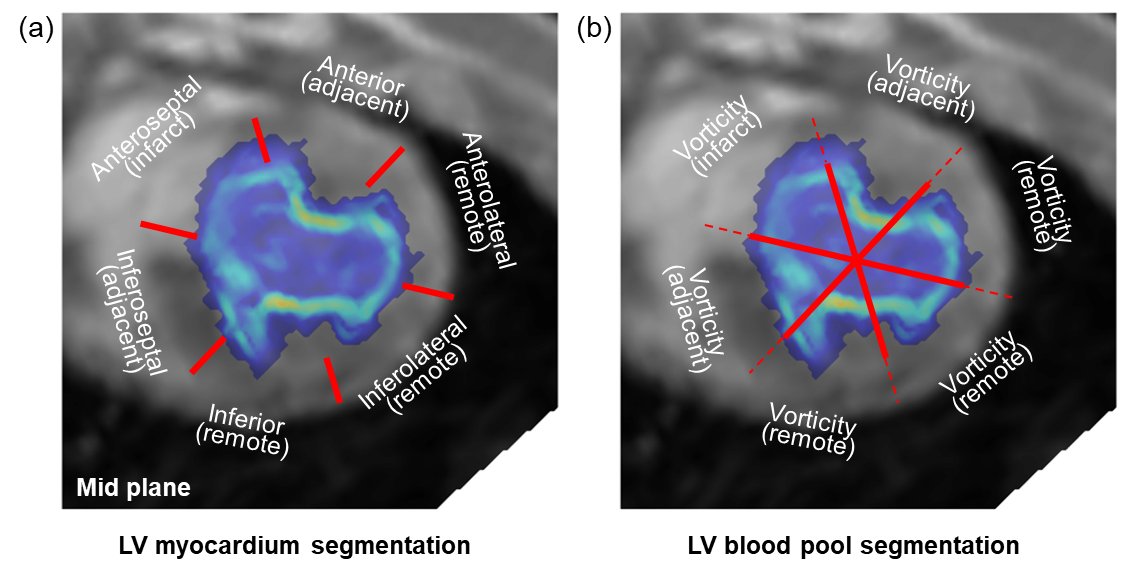


Supplementary Figure S2. Representative segmentation at the mid-ventricular plane. (a) LV myocardial segmentation based on the American Heart Association 16-segment model. (b) LV blood pool segmentation defined by radially extending lines aligned with the myocardial segment boundaries.


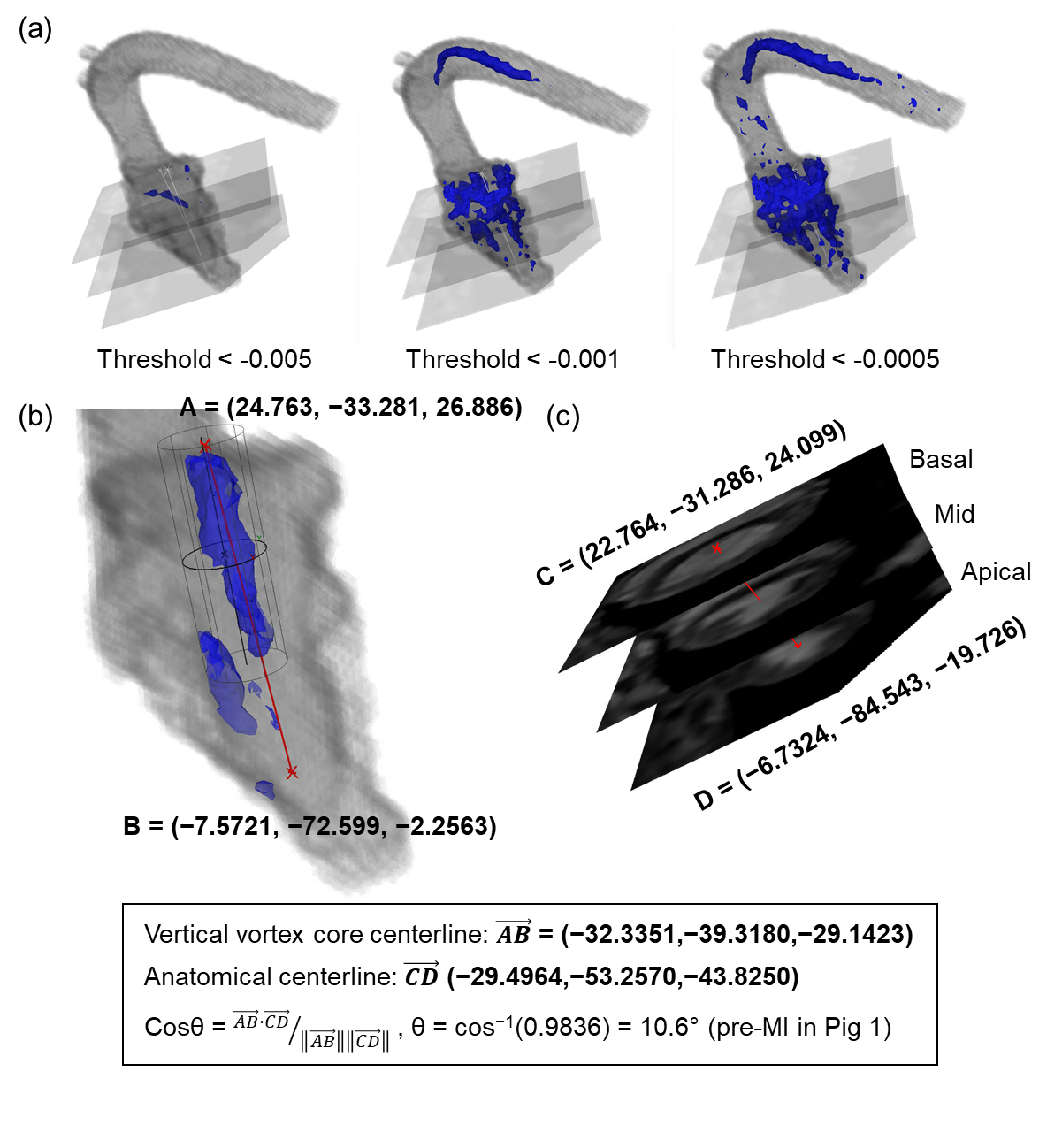


Supplementary Figure S3. Overview of vertical vortex core (VVC) and anatomical centerline extraction. (a) Lambda2-based vortex visualization is highly sensitive to the selected threshold value. Depending on the underlying flow features in each case, the threshold must be optimized to appropriately visualize the VVC. (b) After specifying the threshold value (e.g., −0.0005), vortex structures outside the region of interest are removed, leaving only the VVC. A two-point spline is then generated through the center of the extracted VVC. (c) The anatomical centerline is defined by connecting the centers of the basal and apical planes. This approach is less affected by landmark displacement or left ventricular dilation in the mid-infarcted region.


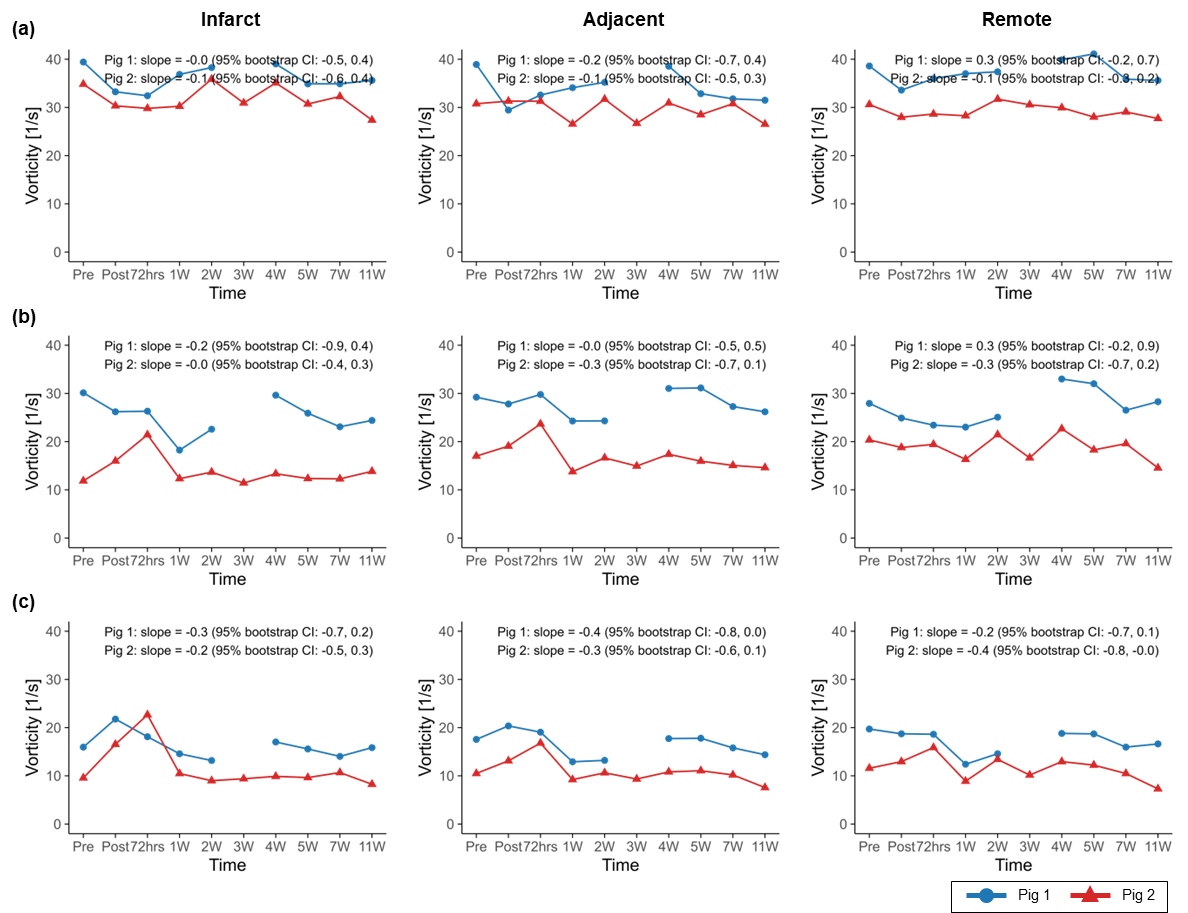


Supplementary Figure S4. Temporal changes in vorticity at the (a) basal, (b) mid, and (c) apical planes across infarct, adjacent, and remote regions during the study time. Temporal trajectories are summarized descriptively, with overall trends characterized using Theil–Sen slope estimates and 95% residual bootstrap confidence intervals. Note that even the infarct regions did not appear to markedly affect intracardiac vorticity within our study period.


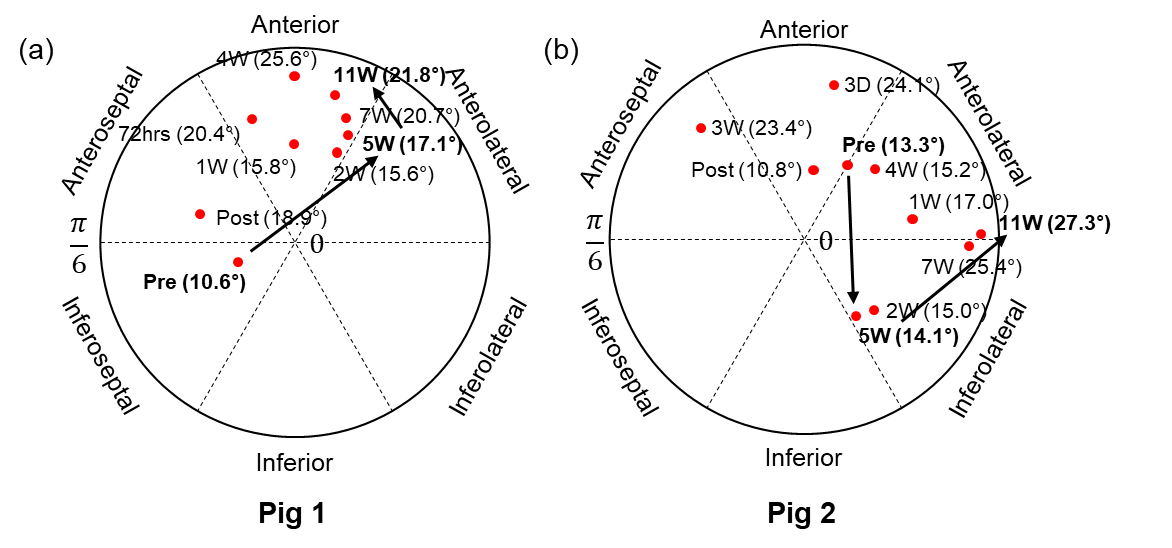


Supplementary Figure S5. Changes in vortex core orientation and angle are visualized for (a) Pig 1 and (b) Pig 2, shown as red dots. The center of each angular plot represents the anatomical reference orientation at the corresponding time point. Key time points (Pre-MI, 5W, and 11W) are highlighted in bold and connected by black arrows to illustrate the temporal progression of vertical vortex core formation.


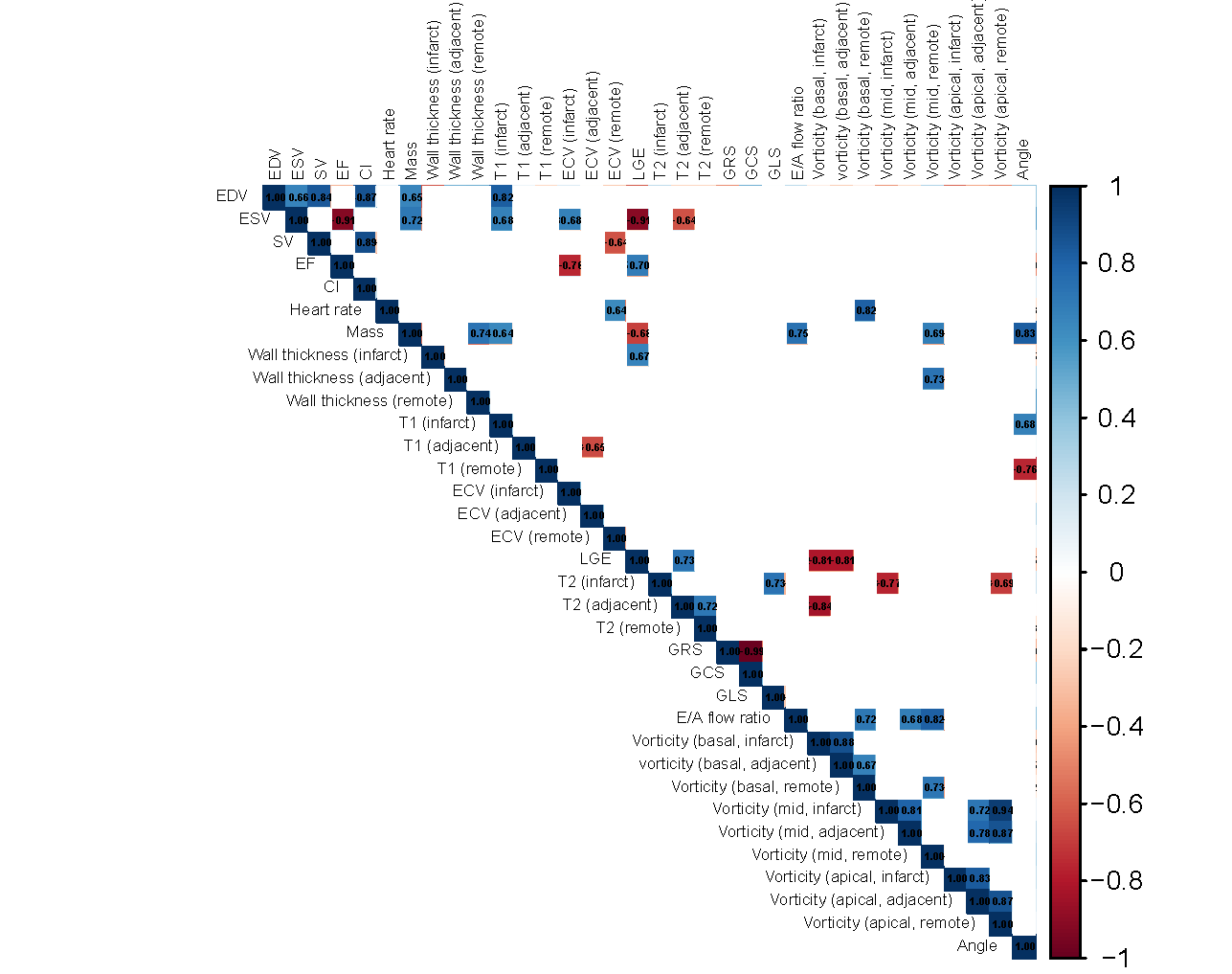


Supplementary Figure S6. Correlation matrix of structural, functional, tissue, and flow-based parameters for pig 1. Color scale represents correlation strength, with blue indicating positive correlations and red indicating negative correlations. Correlation coefficients are shown only when p < 0.05. Pearson’s correlation was used for parametric data and Spearman’s correlation was used for non-parametric data.


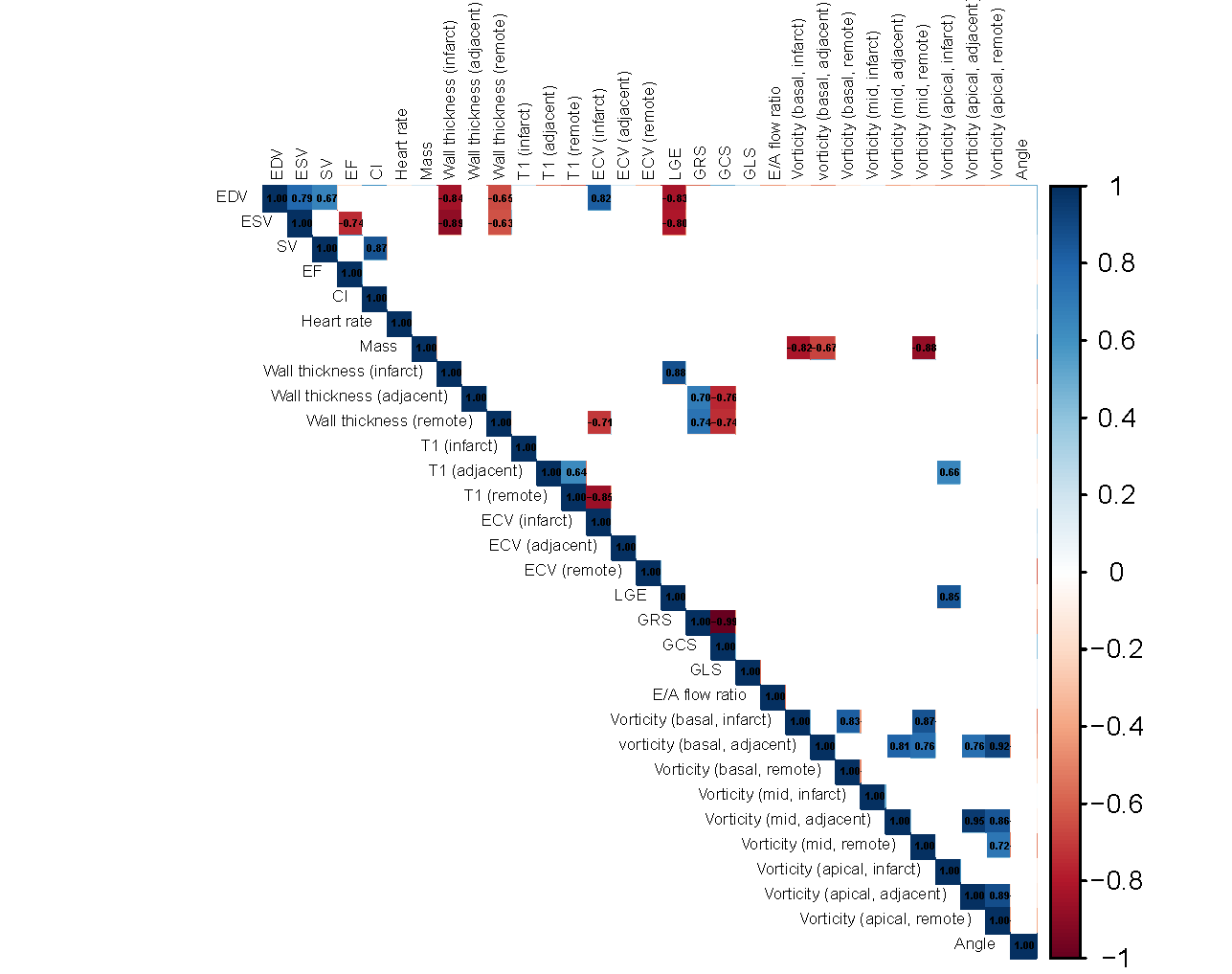


Supplementary Figure S7. Correlation matrix of structural, functional, tissue, and flow-based parameters for pig 2. Color scale represents correlation strength, with blue indicating positive correlations and red indicating negative correlations. Correlation coefficients are shown only when p < 0.05. Pearson’s correlation was used for parametric data and Spearman’s correlation was used for non-parametric data.
